# Supplementary material for: Identification of photoperiod-induced specific miRNAs in the adrenal glands of Sunite sheep (Ovis aries)
Source: Front Vet Sci. 2022 Jul 22;9:888207. doi: 10.3389/fvets.2022.888207 (PMC9354845; doi:10.3389/fvets.2022.888207)
Supplement: Supplementary file 1 [file Data_Sheet_1.ZIP › Supplementary file/miRDeep2 file.zip!/miRDeep2 file/Know_Structure/LP42A1/oar-mir-541.pdf]

miRBase precursor : oar-mir-541
Total read count : 120
oar-miR-541-5p read count : 113
oar-miR-541-3p read count : 7
remaining reads : 0

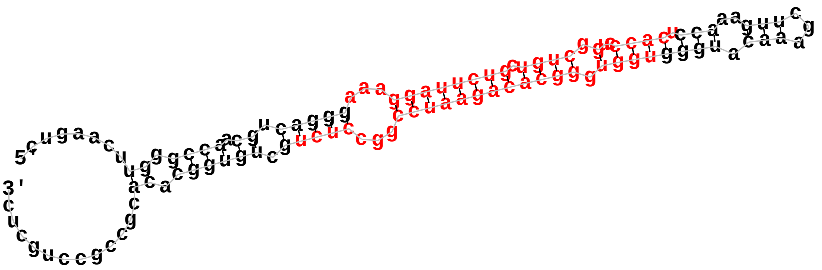

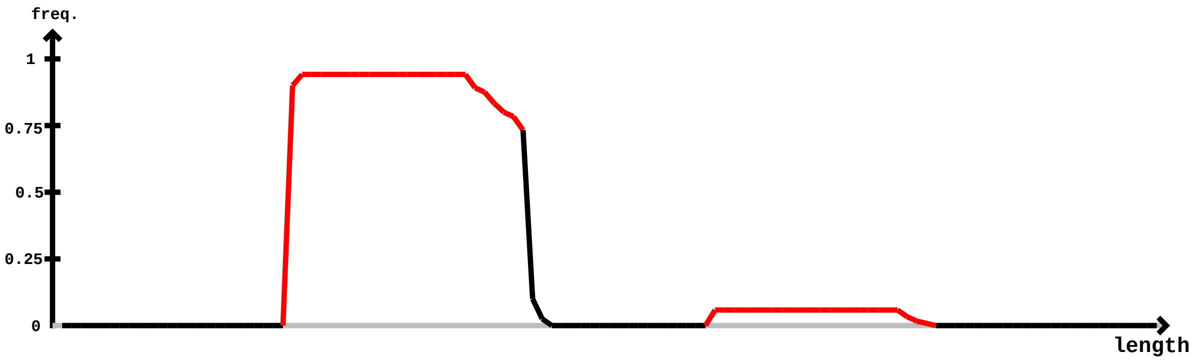


```
                                                                      oar-miR-541-3p
                        oar-miR-541-5p
5'- cugaacuugggccaacgucagggaaaggauucugcugucggucccacuccaaaguucgaaacauggguuggugggcacagaaauccggccucugcuguggcacacgccgccugcuc   -3'    exp
    ........((((..(((.(((((...((((((((.((((.(..((((.(((..(((...))).)))))))).))))))))))))...))))).)))(((....)))))))....    reads      mm         sample
    .......................aaaggauucugcugucggu.........................................................................    6          0          seq
    .......................aaaggauucugcugucgguc........................................................................    2          0          seq
    .......................aaaggauucugcugucggucc.......................................................................    5          0          seq
    .......................aaaggauucugcugucgguccc......................................................................    4          0          seq
    .......................aaaggauucugcugucgguccca.....................................................................    2          0          seq
    .......................aaaggauucugcugucggucccac....................................................................    5          0          seq
    .......................aaaggauucugcugucggucccacu...................................................................    73         0          seq
    .......................aaaggauucugcugucggucccacuc..................................................................    8          0          seq
    .......................aaaggauucugcugucggucccacucc.................................................................    3          0          seq
    ........................aaggauucugcugucggucccac....................................................................    1          0          seq
    ........................aaggauucugcugucggucccacu...................................................................    3          0          seq
    ........................aaggauucugcugucggucccacuc..................................................................    1          0          seq
    ...................................................................uggugggcacagaaauccggc...........................    3          0          seq
    ...................................................................uggugggcacagaaauccggcc..........................    2          0          seq
    ...................................................................uggugggcacagaaauccggccu.........................    1          0          seq
    ...................................................................uggugggcacagaaauccggccuc........................    1          0          seq
```
